# Integrating high-performance computing, machine learning, data management workflows, and infrastructures for multiscale simulations and nanomaterials technologies

**DOI:** 10.3762/bjnano.15.119

**Published:** 2024-11-27

**Authors:** Fabio Le Piane, Mario Vozza, Matteo Baldoni, Francesco Mercuri

**Affiliations:** 1 DAIMON Lab, CNR-ISMN, Bologna, via Gobetti 101, Italy; 2 Department of Computer Science and Engineering, University of Bologna, Bologna, Via Zamboni 33, Italy; 3 Department of Control and Computer Engineering, Polytechnic University of Turin, Turin, Corso Duca degli Abruzzi 24, Italy

**Keywords:** artificial intelligence, high-performance computing, HPC, machine learning, materials modelling, multiscale modelling, nanomaterials, semantic data management

## Abstract

This perspective article explores the convergence of advanced digital technologies, including high-performance computing (HPC), artificial intelligence, machine learning, and sophisticated data management workflows. The primary objective is to enhance the accessibility of multiscale simulations and their integration with other computational techniques, thereby advancing the field of nanomaterials technologies. The proposed approach relies on key strategies and digital technologies employed to achieve efficient and innovative materials discovery, emphasizing a fully digital, data-centric methodology. The integration of methodologies rooted in knowledge and structured information management serves as a foundational element, establishing a framework for representing materials-related information and ensuring interoperability across a diverse range of tools. The paper explores the distinctive features of digital and data-centric approaches and technologies for materials development. It highlights the role of digital twins in research, particularly in the realm of nanomaterials development and examines the impact of knowledge engineering in establishing data and information standards to facilitate interoperability. Furthermore, the paper explores the role of deployment technologies in managing HPC infrastructures. It also addresses the pairing of these technologies with user-friendly development tools to support the adoption of digital methodologies in advanced research.

## Introduction

**Figure 1:**
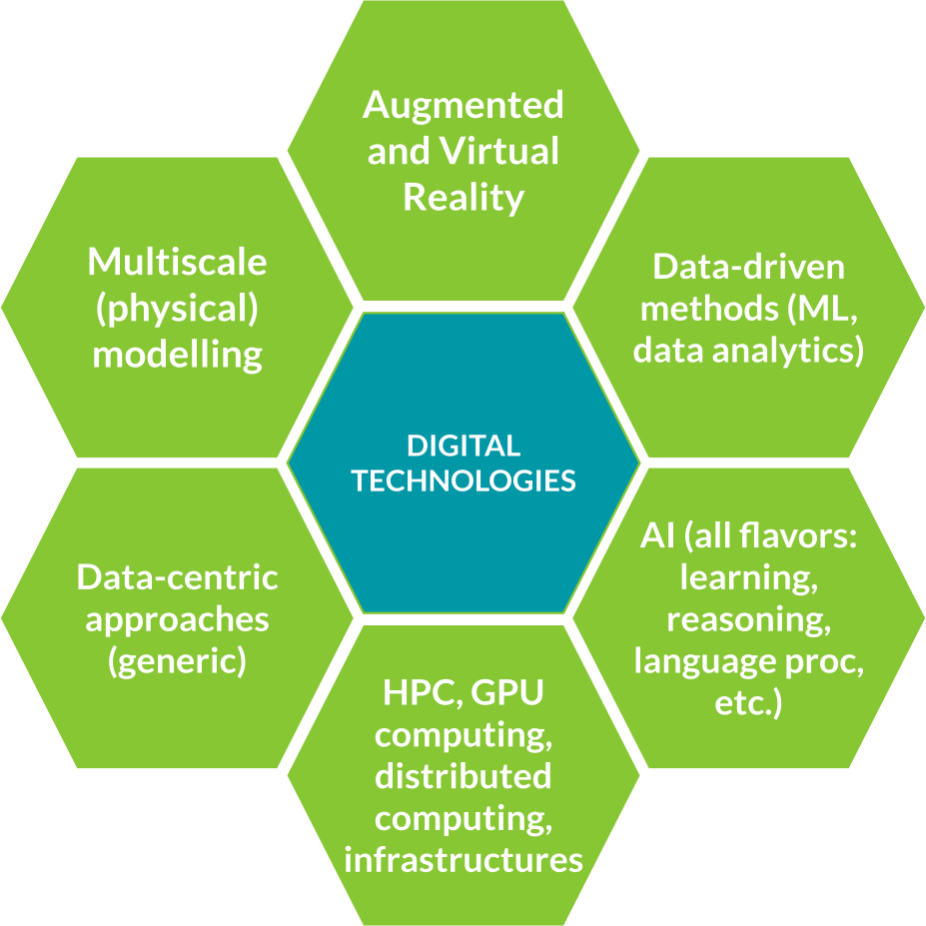
Main digital technologies for materials innovation.

Digital technologies have ushered in a new era of materials science, enabling unprecedented advancements in the design, characterization, and optimization of materials. By leveraging computational modelling and simulation, researchers can simulate and predict properties and behavior of materials with remarkable accuracy, explore a vast design space, and predict the properties and performance of materials before they are synthesized [1–3]. This approach enables the discovery of materials with, for example, improved mechanical strength, enhanced thermal conductivity, superior electrical properties, or other tailored characteristics. Simulations provide crucial insights at different time and length scales, from atomic and molecular-level interactions to the macroscale, that govern the structural, mechanical, and thermal properties of materials [4–5]. More recently, data-driven approaches, such as machine learning (ML) and artificial intelligence (AI), are revolutionizing materials research by extracting valuable patterns and correlations from vast amounts of experimental and computational data [6–9]. These approaches enable researchers to uncover hidden relationships between composition, structure, morphology, processing, and properties, accelerating the discovery of novel materials with tailored functionalities and enabling the identification of patterns and trends. Moreover, high-throughput computational screening allows for the rapid evaluation of extensive material libraries, providing researchers with a systematic and efficient approach to identify promising candidates for specific applications [10]. In addition to materials design, digital technologies can enhance the characterization and understanding of materials. Advanced imaging techniques, coupled with computational analysis, enable researchers to examine the microstructure and behavior of materials at unprecedented resolutions [11–13]. This aids in the understanding of fundamental properties and the identification of structure–property relationships. The integration of digital technologies with experimental techniques also enables real-time monitoring and control of materials synthesis processes, leading to improved reproducibility and quality control. By combining these digital technologies with integrated data management workflows, materials scientists can, in principle, smoothly organize, share, and analyze large volumes of materials data, fostering collaboration and enhancing the overall efficiency of materials research. The integration of digital technologies into materials science has, thus, opened up exciting new possibilities for materials design, discovery, and innovation [14]. New, fully digitalized research directions for materials development are therefore emerging at the convergence of a broad range of advanced digital technologies (Figure 1).

One significant area where these technologies can have a profound impact is in the design and development of advanced nanomaterials [15–16], where the relationship between structure and morphology at different scales, processing, and resulting properties is particularly intricate. The steady and recent advances in hardware and software technologies have propelled materials development in the field. On the hardware front, the continuous improvement of high-performance computing (HPC) systems has enabled researchers to tackle complex computational challenges with greater speed and efficiency. The availability of powerful processors, increased memory capacity, and enhanced parallel computing architectures has significantly accelerated materials simulations and modelling [17]. In parallel, software technologies have undergone remarkable advancements. ML frameworks and algorithms have evolved to handle large and diverse datasets, enabling the extraction of valuable insights from materials data [6]. Additionally, software advancements have facilitated the integration of different computational models, enabling multiscale simulations of materials across a broad range of length and time scales [4,18]. Furthermore, the development of user-friendly interfaces and visualization tools has improved the accessibility and usability of these advanced hardware and software technologies [19–20].

In parallel to the use of large-scale computing infrastructures, consumer-driven off-the-shelf computational technologies have emerged as powerful tools for materials simulations, empowering researchers with accessible and affordable solutions. One notable example is the utilization of consumer graphics processing units (GPUs) for accelerated materials simulations [21–22]. Modern GPUs, originally designed for gaming and multimedia applications, possess immense parallel processing capabilities that can be harnessed for scientific computations. Researchers have successfully leveraged GPUs to accelerate computationally intensive simulations, such as molecular dynamics and quantum chemistry calculations [23–24]. Even more significant has been the impact of GPU computing on AI. GPUs are inherently designed for parallel processing, making them exceptionally well-suited for the demanding calculations and massive data throughput required in AI tasks. Accordingly, GPUs are nowadays considered the most efficient technological platform for performing AI and data-intensive tasks [13,25]. This has enabled the development of complex models that can process vast amounts of materials data. Another consumer-driven technology that has boosted the digitalization of materials research is cloud computing. Cloud-based platforms provide on-demand access to HPC resources and large databases and infrastructures. Cloud-based infrastructures for materials research offer scalability, flexibility, and accessibility, empowering researchers to collaborate, analyze data, and perform simulations more effectively [14]. The application of cloud computing to materials research include the use of materials data repositories (e.g., Materials Project [26] and NOMAD [27]), HPC clouds (including commercial providers), materials simulation platforms (Materials Cloud [28]), collaborative research environments (ResearchGate Labs [29], Mendeley Data) and other services for AI, data analytics, visualization, and training. Cloud platforms have also been used to perform simulations in the materials science domain [30] and to perform automated data analysis [31]. However, the power of cloud computing is being enforced even in other computationally intensive domains such as climate modelling [32], further highlighting how this computing paradigm can be a crucial enabler for higher-scale simulations and modelling activities. Moreover, the continuous development of efficient open-source software packages has boosted the field of materials simulations. Advanced tools for the simulation of materials across a broad range of scales, such as Quantum ESPRESSO [33], LAMMPS [34], GROMACS [35], and OpenFOAM [36], implement complex simulation algorithms, making it easier for researchers to perform complex simulations without extensive programming knowledge. The open-source nature of these packages encourages community contributions, fostering a collaborative environment and driving continuous improvement in materials simulation capabilities. Additionally, consumer-driven technologies like virtual reality (VR) and augmented reality (AR) have shown promise in materials visualization and design. VR and AR platforms offer immersive and interactive experiences, enabling researchers to visualize complex material structures, analyze properties, and manipulate models in real time. These technologies enhance the path towards the development of new materials, facilitating informed decision-making and accelerating the design of novel materials with desired characteristics [37–39]. These key technologies can enable the disruptive potential of digital technologies in materials development by addressing aspects related to both predictivity and automation. The integration of multiscale physical and data-driven modelling of materials can support the prediction of materials properties and the design of novel materials and processes. In addition, digitalization also enables the uptake of automation in materials development. Beside the implementation of automation and robotics in the development, synthesis, and characterization of materials, automation in modelling has emerged as a powerful approach to streamline and enhance the efficiency of computational studies. By leveraging digital technologies and advanced algorithms, researchers can automate different aspects of the materials modelling process, from data generation to model selection and parameter optimization [7,40–41]. Furthermore, automation enables the integration of experimental data with computational models, facilitating the calibration and validation of models and providing a more comprehensive understanding of materials behavior [10]. The automation of various modelling tasks, such as data preprocessing, model generation, and parameter optimization, through the use of advanced algorithms and software tools, streamlines computational workflows and minimizes manual effort. This automation not only improves efficiency but also enhances reproducibility and reduces the potential for human error.

User-friendliness of software platforms and frameworks used for materials modelling tasks has also significantly improved in recent years. Ready-to-use software packages provide pre-implemented algorithms and methods, eliminating the need for researchers to develop complex simulation platforms from scratch. The availability of software platforms and packages and interfaces enables a more efficient translation of scientific and technological questions into simulation and modelling workflows [42–43]. Additionally, these tools often come with pre-built databases, libraries, and visualization capabilities, further enhancing their usability and efficiency.

In this work, we outline different aspects of data-intensive digital and integration technologies, outlining their role as key enablers for the realization of digital twins (DTs) in the context of materials and nanomaterials development. We will also showcase some of the work carried out towards these goals, illustrating the main principles behind the development of tools and approaches. The paper is structured as follows: The first section revolves around data-centric approaches for materials development, emphasizing the pivotal role of data; the second section is about the realization of digital twins of nanomaterials, elucidating conceptualization and implementation; the third section is about key enabling digital technologies in materials development, highlighting a fully digital, data-centric approach through the integration of HPC and ML technologies; in the fourth section, we outline the role of semantic technologies for the management of data and information within materials development; in the fifth section we describe infrastructures supporting data-centric workflows, covering common development tools for research on nanomaterials, workflow building tools, and deployment strategies such as virtualization and containerization; finally, we describe a typical application scenario featuring most of the approaches and technologies discussed in the paper.

## Data-centric approaches for materials development

Data-centric approaches are revolutionizing conventional materials development pipelines by streamlining and informing the entire workflow. Traditionally, materials development relied heavily on experimental characterization and trial-and-error methods, which can be time-consuming and resource-intensive. However, with the rise of digital technologies, data-centric approaches have emerged as a more efficient and effective alternative [6,8,44–45].

**Figure 2:**
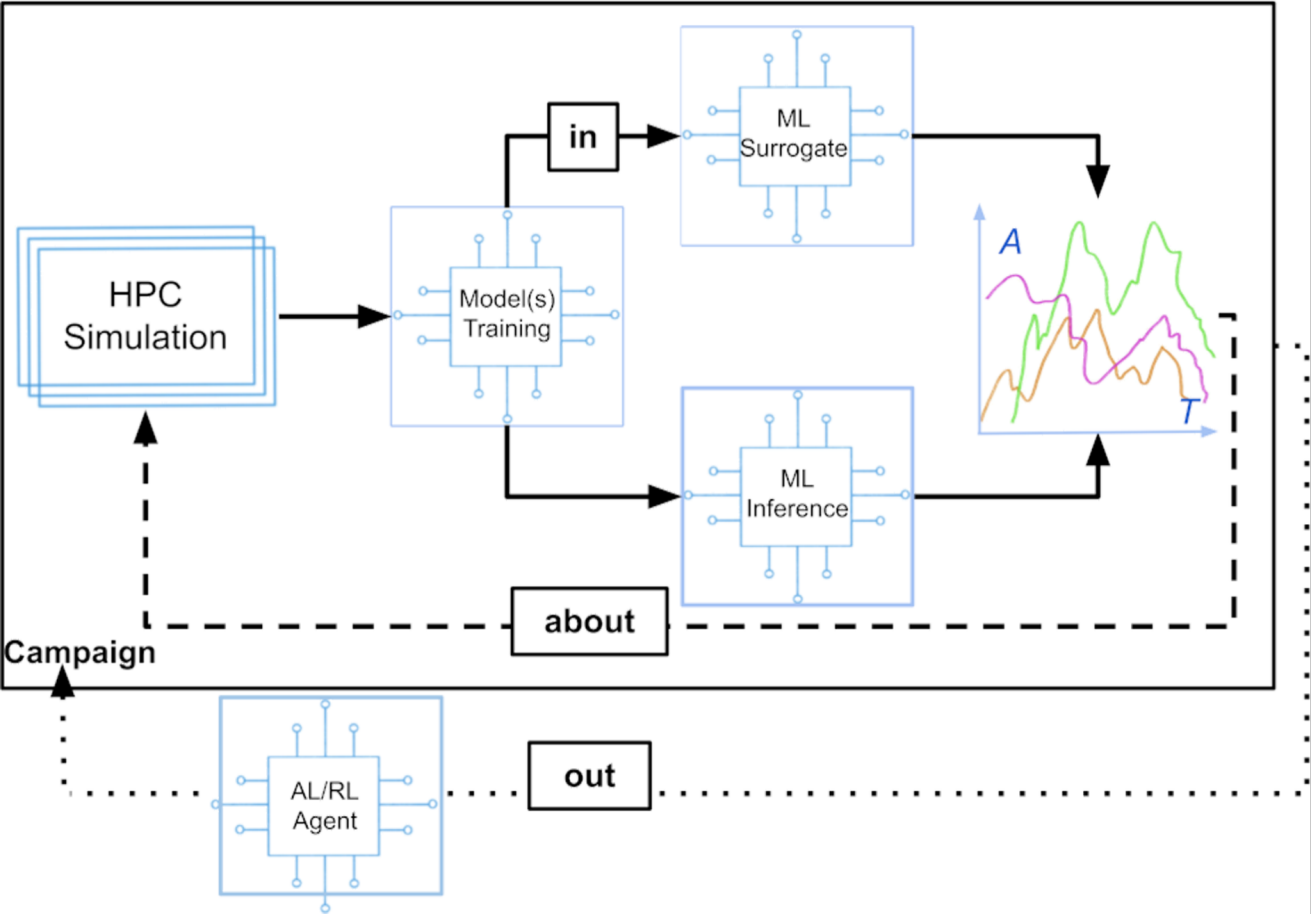
Merging ML with HPC infrastructures can be done in three different ways: ML-in-HPC uses AI/ML surrogate models to replace simulations, ML-about-HPC complements and potentially directs traditional computational tasks, and ML-out-HPC employs high-level AI/ML algorithms, such as active learning or reinforcement learning, to dynamically control the overall workflow. ML-in and ML-about directly produce output for analysis, while ML-out drives this production. This figure was reprinted from [49] by Jha, S.; Pascuzzi, V.; Turilli, M., "AI-coupled HPC Workflows" in "Artificial Intelligence for Science: A Deep Learning Revolution", Choudhary, A.; Fox, G.; Hey, T. Eds. p. 515–534, Copyright 2023 World Scientific Publishing. It is used with permission from World Science. This content is not subject to CC BY 4.0.

The role of data-centric approaches in the development of materials, typically occurs at three levels, that are related to (i) intrinsically digital data, (ii) experimental data from high-throughput setups, and (iii) complex and integrated datasets. Approaches based on intrinsically digital data, such as those originating from virtual systems, digital twins, computational modelling, HPC, edge computing, and Internet of Things, can, in principle, be directly integrated within data-centric frameworks. As we will see later on, however, the issues related to data integration are also relevant in this case. The analysis and elaboration of data obtained from high-throughput experimental techniques, such as signals and images, have been greatly enhanced by digital technologies, enabling researchers to extract valuable insights and drive materials development [12]. High-throughput experimental methods generate vast amounts of data, which require efficient analysis techniques to uncover meaningful patterns and relationships. Digital technologies provide advanced algorithms and tools to process and interpret these data, enabling researchers to extract quantitative and qualitative information [3,11,46–47]. The integration of data from high-throughput experiments with computational modelling and simulation further enhances the understanding of materials properties and behavior. By combining experimental and computational data, researchers can validate and refine models, improving their accuracy and predictive power [48]. The analysis and elaboration of complex and integrated datasets that combine simulation data with data flows from experiments and measurements have been significantly enhanced by digital technologies. These datasets offer a comprehensive and holistic perspective on materials behavior, enabling researchers to gain deeper insights and make informed decisions. Through the integration of simulation data with experimental measurements, researchers can validate and refine computational models, improving their accuracy and reliability. Advanced data analysis techniques, such as statistical analysis, machine learning, and data fusion methods, enable the integration and interpretation of diverse datasets. By applying these techniques, researchers can uncover correlations, extract meaningful features, and reveal hidden patterns within these complex datasets. Additionally, digital technologies facilitate the visualization and interactive exploration of integrated datasets, allowing researchers to visualize and comprehend intricate relationships between different variables and parameters [24]. This integrated data analysis approach fosters cross-disciplinary collaboration, facilitates knowledge transfer, and enhances the overall understanding of materials properties and behavior. By leveraging the power of digital technologies, researchers can accelerate materials research, streamline materials design processes and foster scientific breakthroughs. A depiction of the interplay between this different technologies and a potential resulting workflow is depicted in Figure 2.

The implementation of digital strategies for materials/nanomaterials development faces several key challenges that must be addressed for successful integration. One of the main issues is the availability and quality of data. Digital strategies heavily rely on data from various sources, including experimental measurements, simulations, and literature databases. However, ensuring the accessibility, reliability, and interoperability of data remains a significant hurdle. Standardization efforts and data sharing platforms are essential to promote cohesive integration and enable effective collaboration among researchers [14,50]. Additionally, the computational infrastructure required to support digital strategies poses a challenge. Accessing and maintaining HPC resources and advanced software tools can be costly and may require specialized expertise. Efforts to enhance the accessibility and affordability of HPC resources, along with user-friendly software interfaces, can help overcome these challenges [19,42–43]. Moreover, the integration of experimental and computational data presents a significant hurdle. Aligning experimental protocols and data formats with computational frameworks is crucial for effective integration and accurate prediction of materials properties. Data security and privacy are also important considerations, requiring robust security measures and adherence to data privacy regulations. Establishing secure data management practices and implementing encryption techniques can help safeguard intellectual property and confidential information [51–52]. Furthermore, the skills and training needed to leverage digital strategies are crucial. Researchers and practitioners need to acquire expertise in computational modelling, data analytics, and relevant software tools. Investing in education and training programs can empower the workforce with the necessary skills to effectively utilize digital strategies in their research endeavors. By addressing these main issues, the implementation of digital strategies can unlock new opportunities and drive advancements in materials and nanomaterials development.

**Figure 3:**
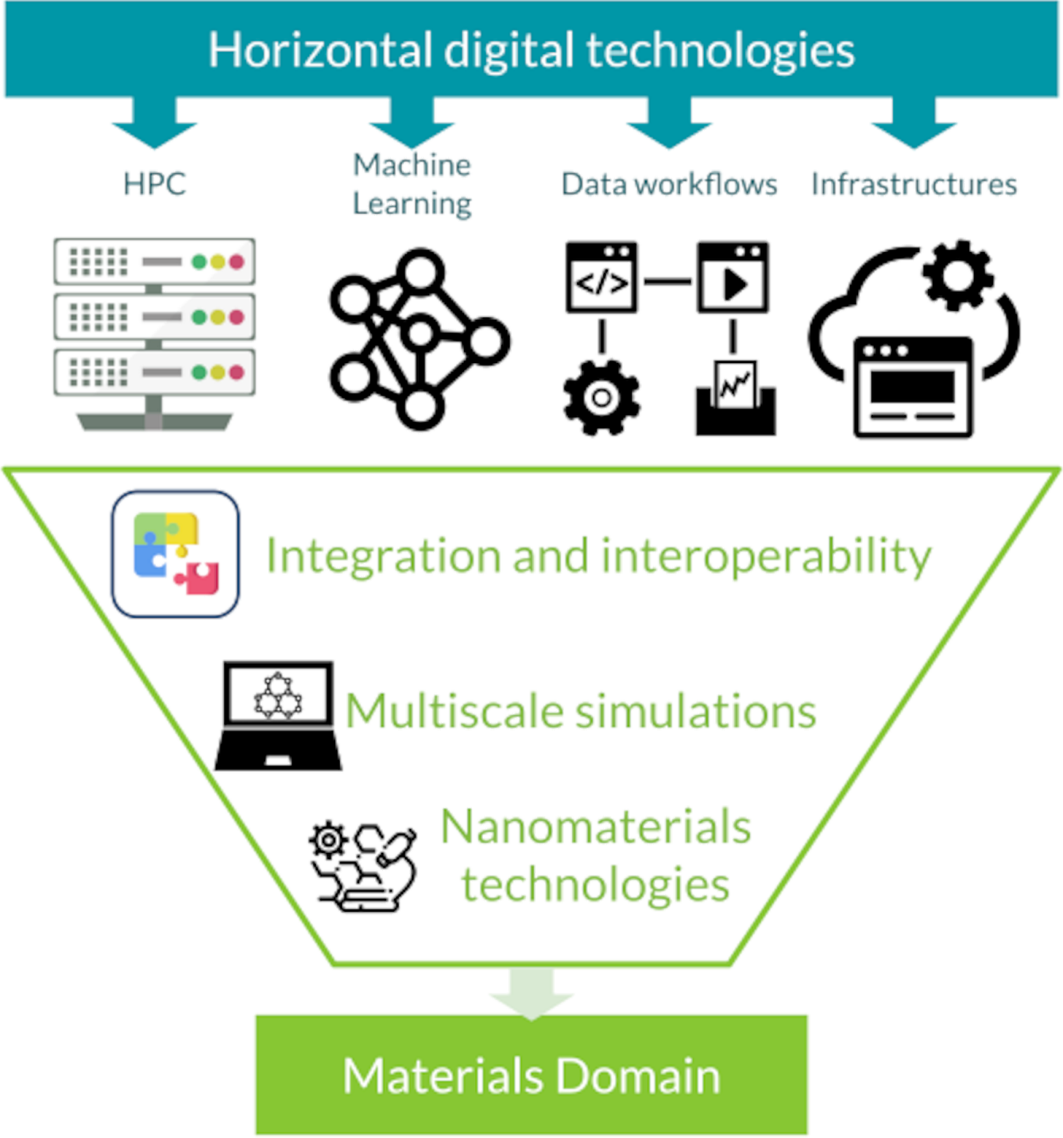
The funnel for the convergence of a manifold of digital technologies towards the materials domain. The included icons are accredited as follows: The HPC icon is from https://www.svgrepo.com/svg/484996/server-network-part-2 under the CC0 License; the machine learning icon is from https://www.svgrepo.com/svg/447866/ai-mi-algorithm under the Public Domain License or CC0 License; the data workflows icon is from https://www.svgrepo.com/svg/7371/data-flow-chart under the CC0 License; the infrastructure icon is from https://uxwing.com/web-service-icon/. This content is not subject to CC BY 4.0; the integration icon is from https://www.svgrepo.com/svg/439194/integration-testing under the MIT License (see https://www.svgrepo.com/page/licensing/#MIT), by Andreas Mehlsen. This content is not subject to CC BY 4.0; the simulation icon is from https://www.svgrepo.com/svg/165724/science-symbols-on-computer-screen under the CC0 License; the nanomaterials technologies icon is from https://www.svgrepo.com/svg/304458/cells-molecule-science-biology-microscope-lab under the CC0 License.

One of the challenges in implementing digital strategies for materials/nanomaterials development lies in translating high-end technologies into specific and narrow research domains. While digital technologies offer tremendous potential, their application in specific research domains requires careful adaptation and customization. Each research domain has its unique requirements, experimental techniques, and data formats, which may not readily align with existing digital tools and frameworks. Translating high-end technologies to these specific domains involves developing domain-specific models, algorithms, and data processing pipelines that cater to the specific needs and constraints of the research area. This requires interdisciplinary collaboration between materials scientists, domain experts, and computational researchers to identify the most relevant and impactful digital technologies, adapt them to the specific research domain, and validate their applicability. Additionally, effective communication and knowledge exchange between different research communities are crucial to ensure a logical integration of digital technologies into specific research domains. By addressing the challenge of translating high-end technologies into narrow research domains, the full potential of digital strategies can be harnessed to accelerate materials discovery and development in targeted areas. For an example of the process that lead from horizontal technologies to a vertical integration to the materials science domain see Figure 3.

**Figure 4:**
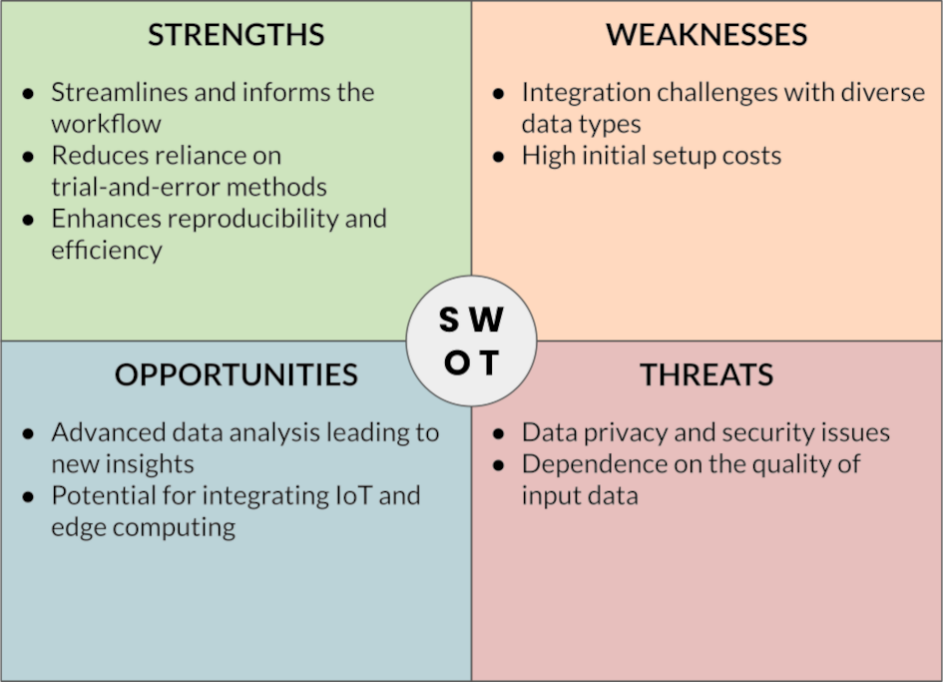
SWOT analysis of data-centric approaches in materials science.

The successful implementation of digital strategies for materials/nanomaterials development relies on the crucial role of “translators” who bridge the gap between domain-specific researchers and digital technology experts. Translators should ideally possess a deep understanding of both the research domain and the capabilities of digital technologies, acting as intermediaries, facilitating effective communication, collaboration, and knowledge exchange between the two groups. Translators potentially play a pivotal role in identifying the specific needs and challenges of the research domain and articulating them to digital technology experts, supporting the translation of domain-specific requirements into technical specifications, and enabling the development of tailored digital solutions. Likewise, translators interpret the capabilities and potential of digital technologies to domain experts, showcasing how these technologies can address their research questions and enhance their workflows. By serving as a liaison, translators ensure that digital strategies are effectively applied in materials/nanomaterials development, leading to more informed decision-making, accelerated discovery, and innovation. Figure 4 summarizes the key point of this sections through a SWOT (“Strengths, Weaknesses, Opportunities, Threats”) analysis.

### Towards a digital twin of nanomaterials

Enabling a “digital twin” of nanomaterials is a critical aspect of digital strategies for materials/nanomaterials development [16]. A digital twin represents a virtual replica of a physical material, capturing its properties, behavior, and performance in a digital form. Creating a digital twin involves integrating various types of data, such as experimental measurements, simulation results, and materials databases, into a unified model. This digital representation enables researchers to explore and analyze materials in a virtual environment, providing insights that would otherwise require extensive and time-consuming experimental testing [53–54]. The digital twin serves as a powerful tool for predictive modelling, optimization, and design of materials, allowing researchers to assess performance under different conditions, predict degradation mechanisms, and optimize material properties. It also facilitates virtual experimentation, reducing the need for costly and resource-intensive physical trials. The development of digital twin frameworks requires interdisciplinary collaboration between materials scientists, data scientists, and computational experts to ensure accurate representation and reliable predictions. By enabling a digital twin of materials, digital strategies offer a transformative approach to materials development, unlocking new avenues for innovation and accelerating the design and optimization of advanced materials.

**Figure 5:**
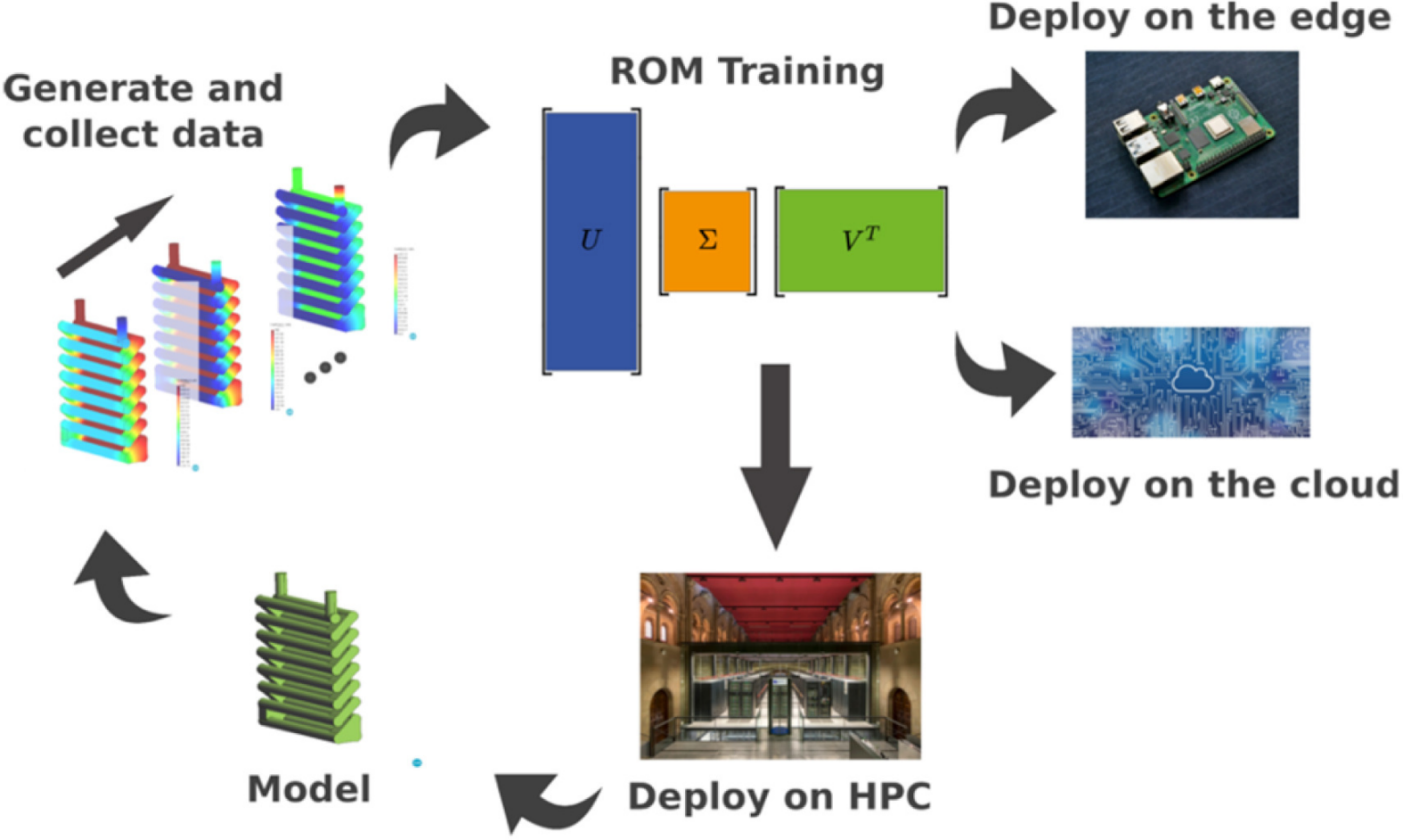
Main building blocks for a workflow comprising data collection, ML model training, and deployment on cloud and HPC cluster. This is a virtuous cycle where each step leads to the next one and then back to first. This figure was published on Future Generation Computer Systems, vol. 134, by J. Ejarque, R. M. Badia, L. Albertin, G. Aloisio, E. Baglione, Y. Becerra, S. Boschert, J. R. Berlin, A. D'Anca, D. Elia, F. Exertier, S. Fiore, J. Flich, A. Folch, S. J. Gibbons, N. Koldunov, F. Lordan, S. Lorito, F. Løvholt, J. Mací­as, M. Volpe, ”Enabling dynamic and intelligent workflows for HPC, data analytics, and AI convergence”, p. 414–429, Copyright Elsevier (2022) [55]. It is used with permission from Elsevier. This content is not subject to CC BY 4.0.

The concept of a digital twin within the materials domain encompasses the integration of both models and data-driven approaches. It involves linking physical and statistical models to data-driven techniques to create a comprehensive digital representation of materials. This integration enables researchers to benefit from the strengths of each approach, combining the fundamental understanding provided by models with the richness and complexity of real-world data. By linking models with data-driven approaches, the digital twin concept offers a powerful framework for advancing materials research, accelerating materials design, and enabling more informed decision-making in the materials domain. Models provide a mathematical or computational description of the behavior of materials, capturing physical, chemical, and mechanical properties. Data-driven approaches leverage large datasets, including experimental measurements, to extract patterns, correlations, and trends in materials behavior. By combining both model-based and data-driven approaches, a digital twin can encompass the complete picture of the performance of materials under different conditions. This mutual positive feedback between model-based simulations and data-driven methods is depicted in Figure 5.

**Figure 6:**
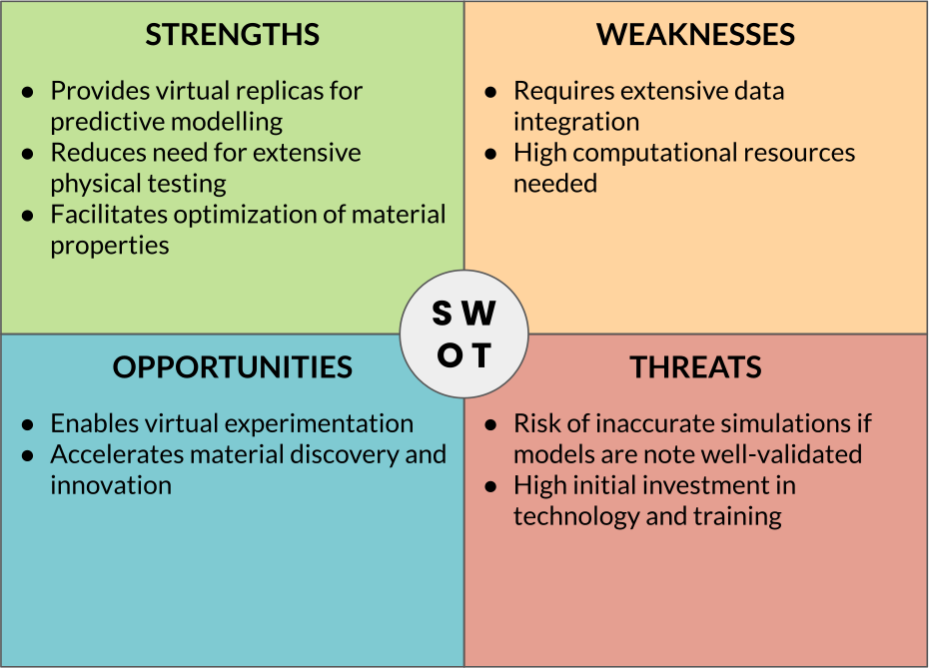
SWOT analysis of digital twin applications for the materials science domain.

In the context of nanomaterials, the digital twin concept involves utilizing models to represent the underlying physics or chemistry of the system, while incorporating data-driven approaches to enhance the accuracy and predictive power of these models. Data-driven techniques provide valuable insights into the complex relationships and interactions within the material, capturing real-world behavior and enabling better calibration and validation of the models. This integration allows researchers to refine and improve the models, making them more accurate and reliable in predicting material properties, performance, and behavior under different scenarios. Physics-based models are built upon fundamental principles and equations, capturing the underlying physics or chemistry of materials. These models describe the interactions between atoms, molecules, or particles, allowing researchers to simulate and predict material properties and behavior at different scales. Physics-based models provide insights into the fundamental mechanisms governing materials phenomena, such as structural changes, phase transitions, and mechanical responses. Empirical models, in contrast, are derived from experimental observations and statistical analyses. These models rely on data collected from experiments and measurements to establish relationships between input variables and desired outputs. Empirical models are often used when the underlying physics or chemistry is not fully understood or when experimental data is abundant. They offer a practical and efficient approach to predict material properties and behavior based on empirical correlations and trends. Data-driven models leverage machine learning and statistical techniques to extract patterns and relationships from large datasets. These models learn from existing data to make predictions or classifications without explicit knowledge of the underlying physical principles. Data-driven models can be trained on diverse datasets, including experimental data, simulation data, and literature data, enabling the discovery of complex relationships and the identification of new material properties or behaviors. The integration of these different types of models is crucial for digital strategies in the development of materials and nanomaterials. Combining physics-based models with empirical or data-driven models allows researchers to benefit from both the understanding provided by fundamental principles and the predictive power of data-driven approaches. The synergy between models enables more accurate predictions, enhances the exploration of materials design space, and accelerates the discovery of novel materials with desired properties. A SWOT analysis of DT applications in the materials development domain is shown in Figure 6.

## Key Enabling Digital Technologies for Materials Development

New paths for materials design and development leverage on digital technologies, merging multiscale physical modelling, data-driven modelling, artificial intelligence, and innovative hardware and software technologies and infrastructures [41,56]. Multiscale modelling constitutes one of the crucial ingredients for linking a physical description of materials to new digital and data-intensive technologies. Accordingly, multiscale modelling has recently gained popularity as the approach of choice in several application domains where the properties of advanced and complex materials are exploited [5,18]. Methods applied in multiscale materials modelling address a broad range of phenomena from the electronic/atomistic to the macroscopic scale. However, the application of comprehensive multiscale models to relevant application scenarios requires a significant amount of computational power at hand, which translates into the need for efficient hardware and software infrastructures and technologies. These requirements often call for the application of HPC and large-scale infrastructures, which require considerable efforts in terms of implementation, management, resources, and power. These strong constraints on infrastructures, competences, and resources constitute a significant barrier for non-specialists or non-academic institutions, for example technological SMEs. Current multiscale approaches also lack a high degree of automation and are more similar to a custom, tailor-made process. The overall modelling workflows can therefore be very time-consuming, in terms of human power required, especially when a broad range of interlinked multiscale models is involved. The lack of consolidated automation workflows turns into a relatively low throughput of multiscale modelling approaches in current scenarios. In recent years, however, we have begun to witness the success of AI and ML for materials development [7,13]. This is particularly evident, for example, in the application of AI-related methods for the prediction of structure–property relationships in materials [6]. Despite these successes in delivering accurate and reliable property predictions based on training datasets, several other extremely powerful applications of AI still need to be fully unraveled. For example, efficient routes for translating the methodologies borrowed from the impressive progress of natural language technologies to the materials domain are just at their early stage. In other words, the application of ML to materials development is largely still at the “empirical” level, that is, supporting the prediction of materials properties within a relatively simple, though numerically very intensive, methodological framework [57]. Largely relying on the property prediction and design sides, data-driven approaches seem to be still quite distant from the concept of a working, comprehensive digital twin of materials. This unstructured approach results in an evident lack of standardization (for example, in the definition of features for materials data across multiscale domains), poor links with specific application domains, and a consequent narrowing of potentially interested communities. Overall, the limitations in the integration between multiscale modelling, AI, and related infrastructures described above, constitute a major obstacle to the implementation of efficient technology transfer pathways for materials development to boost the impact of innovative digital tools to broad socioeconomic sectors. The transfer of knowledge and technology from basic research to applications indeed requires consolidated practices and a sort of robustness of the approaches undertaken. Moreover, the research in the field is still at a lower technology readiness level (TRL) with respect to what is needed for transferring knowledge to real-life applications and scenarios. As stated above, even low-TRL basic research lacks most of the requirements to initiate a path towards standardization and industrial validation. The technical limitations outlined above result in significant issues for technology transfer in the field. These include the lack of industry-grade standards, which results in the adoption of case-by-case approaches and, consequently, in significant requirements in terms of resources. Most application fields and domains also lack consolidated approaches to deal with uncertainties, thus hampering the overall impact of digital tools for materials.

### A fully digital data-centric approach

**Figure 7:**
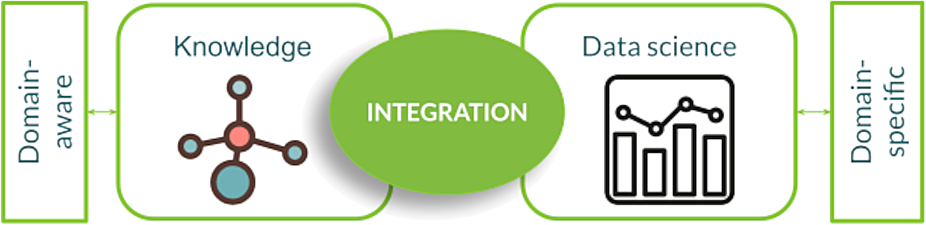
The integration paradigm between knowledge and specific technologies for fully digital data-centric approaches. The included icons are accredited as follows: The molecule icon is from https://www.svgrepo.com/svg/197776/molecule-molecular under the CC0 License. The data science icon is from https://www.svgrepo.com/svg/424587/graph-analytics-business under the CC0 License.

Integration technologies try to tackle the issues outlined above by exploiting the efficiency of digital and data-centric approaches within a specific domain [48,58–59]. In this respect, integration merges tools and technologies within a customized framework and toward a specific goal, thus differentiating from typical consumer-side applications. This approach to integration can therefore be considered at the intersection of knowledge acquired on the domain and data-science specific tools (Figure 7).

**Figure 8:**
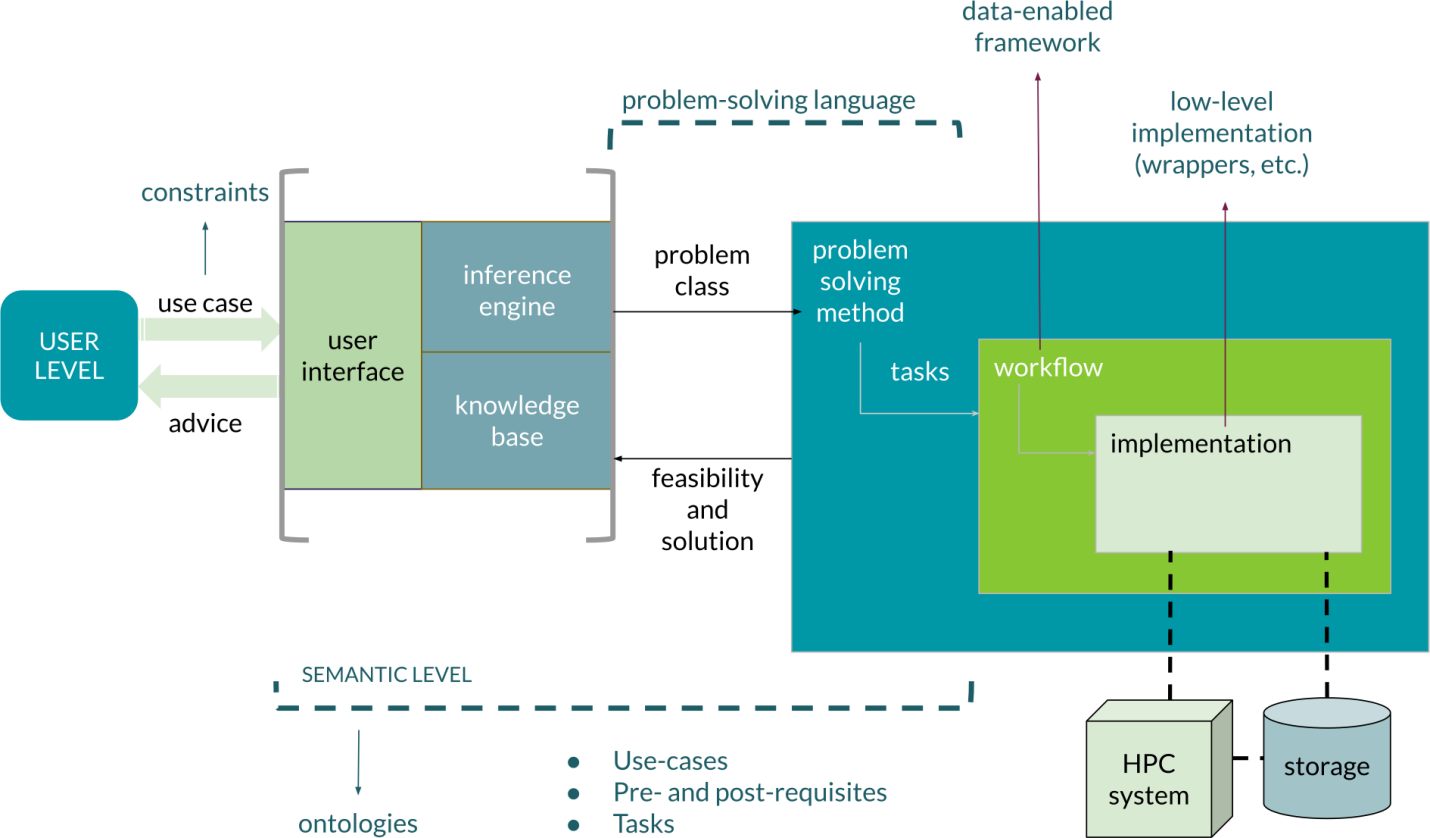
The general architecture of a workflow-oriented data-driven framework for materials development. The components of the framework implement different levels of abstraction depending on their function.

Integration frameworks are implemented as data-centric workflows, where data and information link the components at different abstraction levels [60]. The practical implementation of this kind of integration strategy requires a strong low-level integration technology involving a broad range of components [11]. Robust and efficient software infrastructures are at the core of integration frameworks and should feature a good mix of highly specialized and general purpose tools. Software tools must be paralleled by high-performance hardware infrastructures. These must be able to deal with extremely CPU-intensive and memory-intensive tasks (for example, for dealing with multiscale physical models) and support GPU computing (for deep learning but also for advanced visualization) [61]. The large amount of materials data involved in typical development processes often requires high-performance and high-end storage systems (>100 TB) and high-performance networks and interconnections (100 Gbps and 10 Gbps for local and geographical connections, respectively). On the basis of these conceptual and technical requirements, we can define the generic architecture of a workflow-oriented data-driven high-throughput framework that can be applied to implement a digital multiscale materials development pipeline (Figure 8).

**Figure 9:**
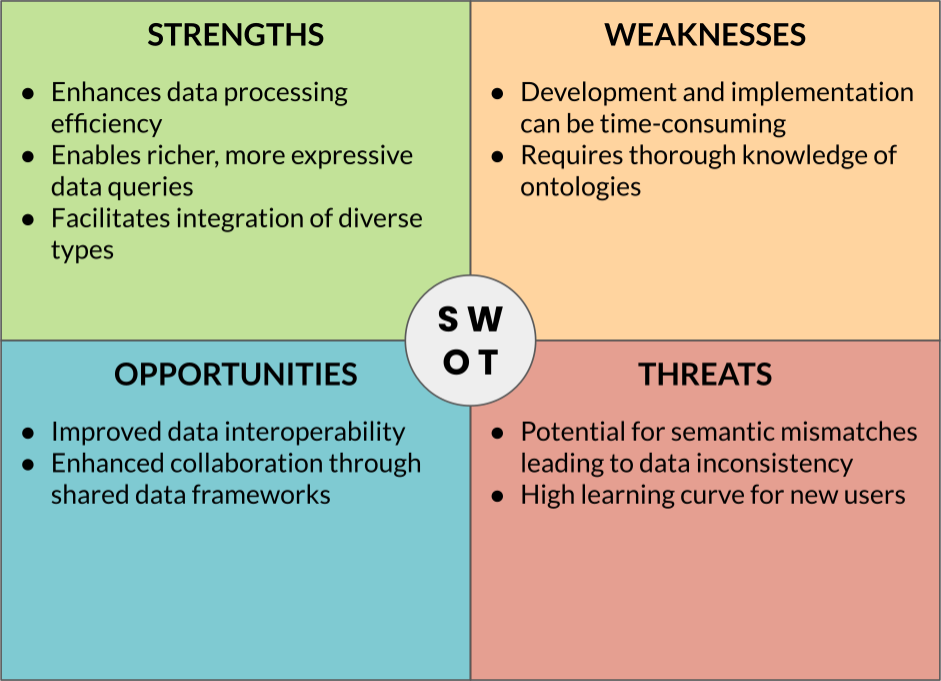
SWOT analysis of semantic technologies in materials science.

The general structure of this framework is based on a set of interfaces and different abstraction layers. General user queries, related to use cases, are translated into tasks and workflows, returning advice and support to decision making [60]. The realization of the framework is based on the interplay between the different levels of abstraction and the corresponding implementation. At the higher abstraction level, semantic technologies constitute a very powerful approach to represent knowledge. This level of abstraction connects high-level information across the framework, guaranteeing consistency from the formulation of queries to the definition of tasks. Ontologies, in particular, constitute an efficient and common way to formally represent knowledge. Accordingly, recent collaborative work has focused on the development of materials ontologies, aiming at developing a shared framework for representing knowledge in the domain [14,50,60,62–63]. The scenarios depicted above require the definition of semantic assets tailored to specific applications of multiscale materials and nanomaterials, thus covering concepts and terms covering both very general purpose domain semantics, typical even in mid-level ontologies, and specific applications. In the ideal scenario, the development of ontologies is therefore driven by workflows designed by end users. A SWOT analysis about the use of semantic technologies in materials science is shown in Figure 9.

With these criteria in mind, we recently worked at the development of MAMBO, the “Materials and Molecules Basic Ontology”.

## MAMBO - the Materials and Molecules Basic Ontology

In the context of the applications of semantic technologies, a solid ontology is the ground of a robust infrastructure. In real-world applications, access to the so-called mid-level domain ontologies is particularly relevant. These are ontologies that enforce more abstract assets defined in higher-level ontologies to formalize knowledge about a more specialized domain (for example, workflows and real-world scenarios). These ontologies serve as the link between general principles and very specific applications. This was the main reason behind the development of an ontology dedicated to molecular materials, that is, MAMBO (the Materials And Molecules Basic Ontology) [64–65]. MAMBO aims to cover areas of knowledge in particular in the domain of molecular materials and nanomaterials. Despite the large amount of work already carried out in the field of ontologies for generic materials and chemical entities, several essential concepts required to deal with the peculiar aspects of molecular materials and nanomaterials are still largely missing.

**Figure 10:**
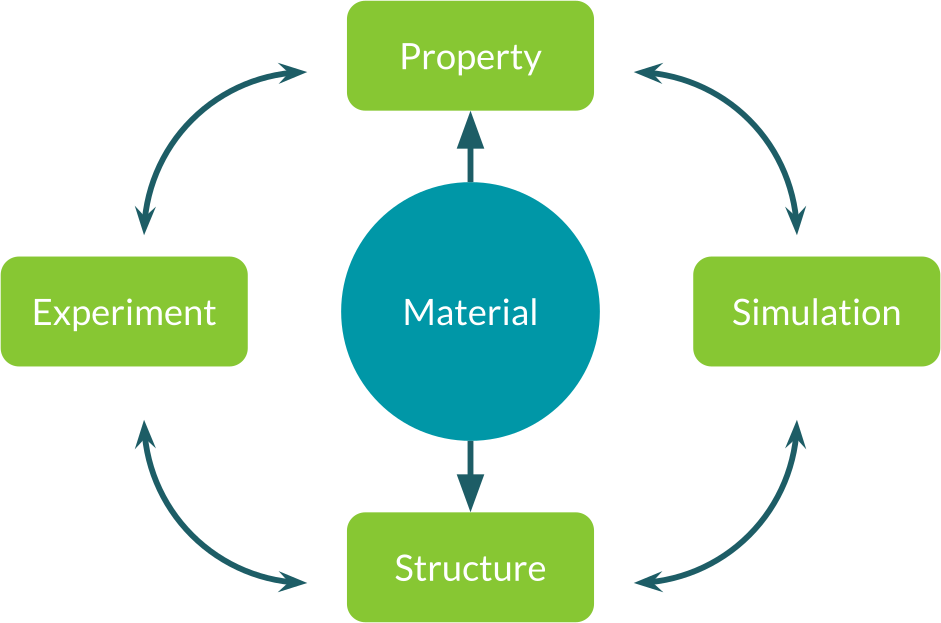
MAMBO main core classes and relationships: the ontology revolves around the concepts of *Material*, *Simulation* and *Experiment*. An object (*Material*) is represented by its structural features (*Structure*) and properties (*Property*), while computational (*Simulation*) and experimental (*Experiment*) workflows are connected through a common interface to *Property* and to *Structure*.

The development of MAMBO followed an hybrid approach mixing top-down and bottom-up processes. To accurately capture the distinct characteristics of concepts integral to the formulation of the MAMBO ontology (both the more general concepts and the more specific ones), we initially constructed a set of qualitative relationships among the identified main terms (such as the concept of “material”, or the concepts of “experiment” and “simulation”). We then refined these concepts, mainly through the results of interviews with domain experts, which have been asked to describe many specific aspects of their research work and activities. Throughout this process, we established the actual classes of the ontology, further enhancing and clarifying their interconnections; with regard to the concepts discussed before, we formally defined classes like *Material*, *Experiment* and *Simulation* for the core of the ontology, and we started to add concepts that are specific to molecular materials, nanomaterials and related domains, such as *MolecularAggregate*. The main core of the ontology can be seen in Figure 10.

As shown in Figure 10, one of the main design choices we made for MAMBO is the representation of both the modelling/simulation activities and the experimental ones using separated classes and hierarchies. This choice allows us to address large parts of the same knowledge base from two different perspectives. From this core, we developed deeper and more specialized hierarchies, which are functional to talk about more specialized concepts such as *Molecule*, *Atom*, and so on. The role of these more specific classes is to give us the possibility to talk about the specific entities and concepts required to describe our research activities and to better define real-world workflows that enforce those concepts in order to link our scientific questions to the final results we need.

Although still in the early development stages, MAMBO proved to be expressive enough to let us represent the knowledge related to computational workflows, using concepts defined in the ontology. This is a first step towards a formal definition of each step of more complex research workflows and for enabling more powerful semantic technologies, where data and the metadata are all encoded using the semantic assets defined in the ontology. This approach leads to a more efficient data processing, as a result of the logical consistency of the definitions used. Data then can act as the glue that make interconnections between different steps of the workflow possible and easier. Moreover, with this kind of representation, we can use as data not only the main information related to a specific workflow, but we can enrich the general knowledge with several other information concerning for example the use of resources or provenance.

### Case-study application of MAMBO

**Figure 11:**
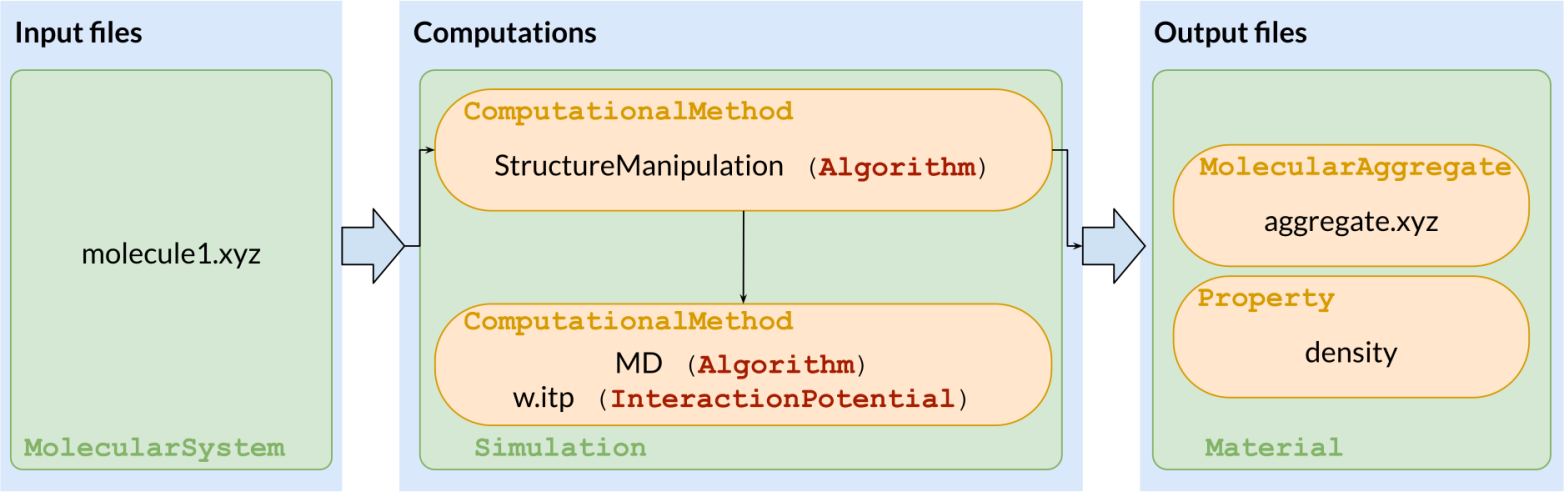
A visual description of the workflow discussed. The first block contains the input files, which are representable as *MolecularSystem* instances as individuals; the second block consists of all the files and software needed to perform the actual simulation; finally, the third block represents the output obtained from the simulation, with information about the structure of the molecular aggregate and the resulting computed density.

The applicability of MAMBO in the organization of knowledge in the target domain was assessed by analyzing simple typical workflows related to R&D for materials and in particular molecular materials. In this section, we will discuss a case study related to the implementation of simulation workflows for investigations of the properties of molecular materials and nanoscale molecular aggregates. To this end, we will use MAMBO classes and relationships that, for the sake of brevity, we cannot introduce here. Interested readers can find more details in [64–65]. The analysis of a case study focusing on simulation workflows, in particular, allows us to define technical requirements and possibly tune the expressiveness of MAMBO in addressing the specific knowledge involved in the description of materials at different scales (from particles to aggregates). Our approach is based on analyzing a general workflow that connects initial information and conditions (pre-requisites) and the final output (post-requisites) of the problem under investigation, further decomposing the problem into tasks and subtasks. The definition of tasks and subtasks and the domain knowledge is organized in terms of the structure provided by MAMBO. Let us first consider a simulation workflow for the evaluation of the physicochemical properties of a molecular aggregate made of identical molecules based on force-field molecular dynamics (MD). While simple, this workflow exhibits the main features of more complex simulations. The consistent representation of this workflow within MAMBO can therefore be instructive of the approach pursued and gives possible hints of the ability to formalize more complex cases. This macrotask can be decomposed into several interconnected computational subtasks, which involve different operations on structured data. From the practical point of view, the overall workflow is generally realized by applying specialized simulation software, which implements specific computational methods, operating on structured input files and producing output files as results. Other operations may require the manipulation of files and data structures. In the case of the considered workflow, we need, for example, input files containing information about the structure of the molecule under study. This information is further processed by specialized software, implementing computational methods, which provide an output in terms of molecular properties. These methods can include, for example, structure manipulation tools (such as simulation box builders) and MD-specific algorithms for equilibrating molecular aggregates under different conditions [66–67]. The workflow produces structured information containing, for example, a snapshot of the structure of the simulated aggregate under the considered conditions and/or derived properties (for example, the computed equilibrium density of the aggregate in kg·m^−3^). A sketch of this workflow is shown in Figure 11.

The decomposition of the workflow sketched in Figure 11 highlights the parallelism between the involved knowledge and instances of MAMBO classes. For example, we can identify the following: (i) The initial information about the molecular system considered is an instance of the *Structure* class, which is linked to the *Material* class via the *has_structure* relationship. In particular, the information pertains to the *MolecularSystem* subclass. (ii) More detailed knowledge on the molecular system considered can be structured in terms of instances of the *Atom* class, which contains information about individual atoms of the molecule. In turn, the position of individual atoms corresponds to instances of the *CartesianCoordinates* class. (iii) Information on the tools for the manipulation of data structure and on MD algorithms can be represented as instances of the *ComputationalMethod* class. (iv) In analogy with the input data, part of the information provided by the workflow can be represented as an instance of the *Structure* class. In particular, the simulated structure of the molecular aggregate is an instance of the *MolecularAggregate* class. (v) The computed property of the molecular aggregate (for example, the computed density) is an instance of the *Property* class.

**Figure 12:**
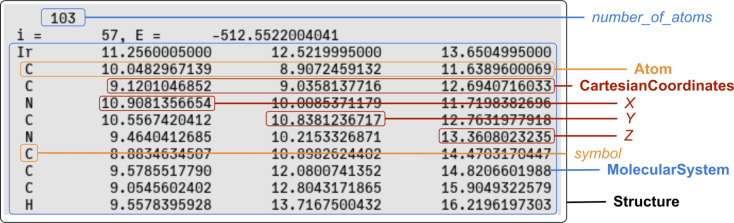
An excerpt of a real-world input file containing structural information about a molecule encoded in the standard *xyz* format. In particular, the file contains information on the Cartesian coordinates and symbols of all the atoms in the molecule and the total number of atoms. Some of the involved MAMBO instances and class attributes are highlighted in different colors. Black: *Structure* instance, blue: *MolecularSystem* instance, orange: *Atom* instance and attributes, and red: *CartesianCoordinates* instance and attributes.

**Figure 13:**
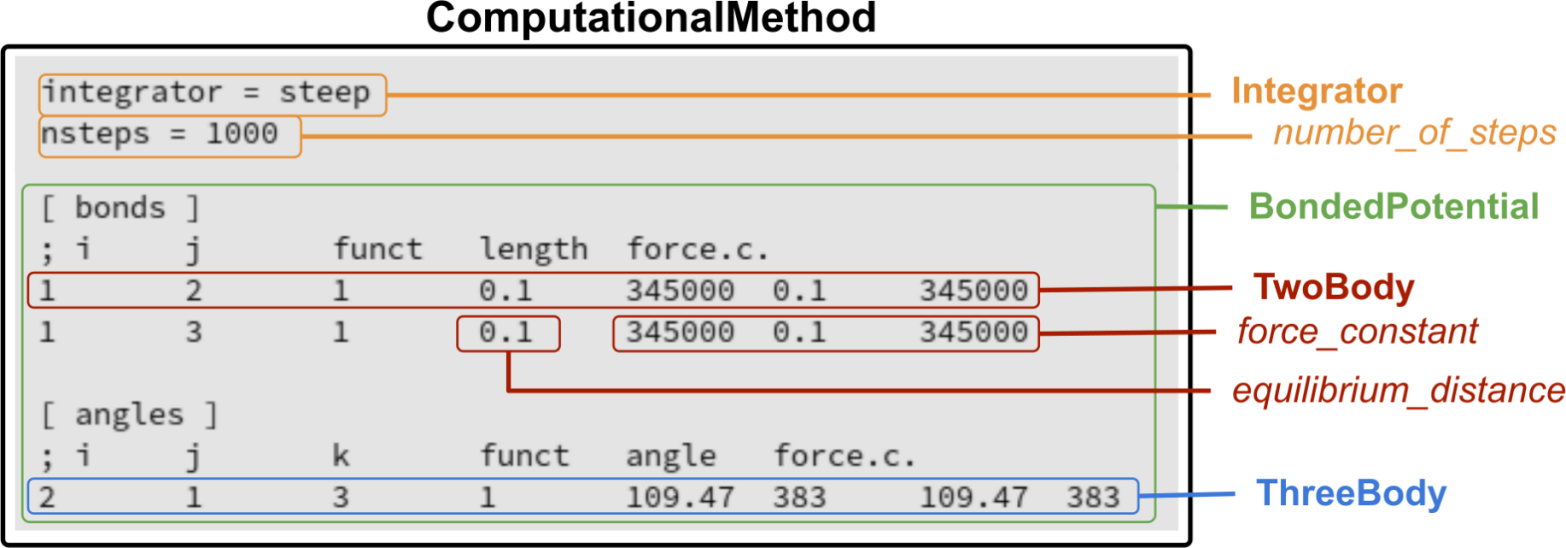
An excerpt of a real-world configuration file containing information about a simulation. This example shows possible encoding in formats used by common software packages for MD simulations (here, a syntax borrowed from the Gromacs [35] format is considered). In particular, the file contains information about the type of *Integrator*, the definition of the interaction potential used in MD simulations (for example, parameters for bonded potential terms, collected by an instance of *BondedPotential*). Involved MAMBO instances and class attributes are highlighted in different colors. Black: *ComputationalMethod* instance, green: *BondedPotential* instance, blue: *ThreeBody* instance, red: *TwoBody* instance and attributes and yellow: *Integrator* instance and attributes.

An example of the parallelism between the structural information on a molecule stored as a file and encoded in a standard format in the context of molecular simulations (*xyz* format) and corresponding attributes of MAMBO classes is shown in Figure 12. A similar example for attributes of classes pertaining to the *ComputationalMethod* class is shown in Figure 13.

The link between the structure provided by MAMBO and the data defining a specific computational workflow can be provided by metadata and/or annotations, which can be implemented in a variety of standard formats [68]. The applicability of MAMBO in the definition of the workflow considered above and defined by exploiting problem-solving methods [69] (competences - input/output, operational specifications and requirements) shows the potential of the proposed approach in the context of specific applications in the materials development pipeline. This approach can be easily extended to more complex systems and processes. The semantic interoperability ground provided by MAMBO in the materials science domain provides the basic components to represent complex workflows in terms of basic and reusable building blocks enabling high-throughput and automated data processing.

## IATA Frameworks

Integrated Approaches to Testing and Assessment (IATA) frameworks constitute another key set of technologies in the context of materials digitalization. IATA tools combine various testing and assessment methods to provide a comprehensive evaluation of materials, including nanomaterials. In particular, IATA frameworks leverage computational models, experimental data, and ML techniques to predict properties and behavior of materials, thus facilitating the integration of diverse data sources and tools to develop predictive models under a structured assessment strategy. Among the broad range of tools available for supporting the development of digital twins of materials and the evaluation of molecular descriptors within an IATA framework, there are the following:

**VMD** (Visual Molecular Dynamics) is a molecular visualization program that provides a platform for the modelling, visualization, and analysis of molecular and biological systems. It is widely used for the development of materials’ digital twins and the calculation of molecular descriptors that can be integrated into ML models [70].

**Enalos NanoInformatics Cloud Platform** is a web-based platform that allows users to design and build nanomaterials. It supports the calculation of molecular descriptors and the integration of these descriptors into ML models for predictive analysis [71]. Moreover, it is tailored to the safe-by-design paradigm, making it an essential tool for future researches [72].

**ASCOT** (an acronym derived from Ag-Silver, Copper Oxide, Titanium Oxide) is a tool for the automated construction and optimization of molecular structures for, as the name suggests, silver, copper oxide, and titanium oxide [73]. ASCOT assists in the generation of high-quality digital twins of materials and the computation of relevant molecular descriptors.

**Nanotube Modeler** is a software tool designed to create three-dimensional coordinates for various nanoscale carbon structures, including nanotubes, nanocones, and fullerenes. The software generates precise *xyz* coordinates for these molecular models. Users can visualize the resulting structures using either the built-in viewer or by exporting the data to their preferred visualization software [74–75].

## Infrastructures for Data

To fortify the foundation given by the robust data structures and metadata that derive from the usage of ontologies, it must be noted how the ability to easily upload and share the resulting data plays a pivotal role. In the realm of contemporary data management, the advent of cloud technologies has emerged as a pivotal catalyst, revolutionizing the infrastructures for data [28]. Cloud technologies represent the most efficient and dynamic means to facilitate the seamless sharing of knowledge across diverse platforms. The inherent scalability, flexibility, and accessibility of cloud-based systems provide researchers and organizations with unprecedented capabilities to store, process, and retrieve vast volumes of data [17]. However, harnessing the full potential of cloud technologies demands a conscientious commitment to deep structuring and restructuring of data. This intricate process involves the precise organization and optimization of information repositories to ensure optimal performance and resource utilization. Consequently, the synergy between cloud technologies and meticulous data structuring heralds a new era in scientific inquiry, empowering researchers to navigate the complex landscape of information with unprecedented efficiency and agility.

### Development tools

In the realm of computational research, the use of local development tools (both on workstations and on HPC facilities) plays a pivotal role in facilitating research, enabling scientists to smoothly transition from theoretical concepts to practical workflows and results. In this section, we are going to highlight some of these tools.

#### The Jupyter ecosystem

In recent years, we have seen the rise of the Jupyter ecosystem, a set of tools developed to make scientific programming easier (even for novices), interactive, and reproducible, while giving the possibility to mix actual code with a markdown text and different media, an approach very akin to that of literate programming [76]. The main component of the Jupyter ecosystem is the Jupyter Notebook. The Jupyter Notebook provides an interactive computing environment that combines code execution, rich text, and multimedia elements into a single document [77]. Scientists can leverage Jupyter notebooks to develop, document, and share computational workflows. These notebooks serve as an interface where theoretical concepts are transformed into executable code, enhancing collaboration and reproducibility in research. We can use notebooks to turn the general concepts and the usual scripts, files, software configurations, and the documents containing technical and scientific explanations into a series of unified files that serve as both the actual executables and the explanatory file. Thanks to the possibility offered by Jupyter notebooks to integrate code with explanatory text (with the rich text rendering capabilities of markdown documents), images, plots, and visualizations in general, researchers can create comprehensive narratives around their computational experiments. This integration fosters a seamless transition from theoretical concepts to practical workflows. Researchers can articulate their thought processes, present results visually, and iterate on their code, fostering a dynamic and iterative research environment. Moreover, thanks to the different media we can integrate inside a notebook and thanks to the possibility to use notebooks for a growing number of programming languages [78], even new researchers with no prior experience with computational tools and HPC as a whole can start to develop their workflows and computational experiments through a friendly, powerful, and intuitive environment.

To make notebooks even more powerful, the Jupyter project introduced a new editor called Jupyter Lab. Jupyter Lab represents the next-generation interface for Jupyter notebooks, offering an actual integrated development environment (IDE) with enhanced features [79]. Its modular architecture allows users to arrange and organize components to suit their workflow preferences, providing a more versatile and customizable experience compared to traditional Jupyter notebooks. Other than the familiar notebook file format and interface, Jupyter Lab offers better filesystem navigation and better visualization capabilities; it also offers the possibility to edit standard text files together with notebooks. Moreover, Jupyter Lab offers real-time collaboration editing capabilities [80], allowing researchers to collaboratively edit their notebooks, meaning that the code, the explanatory text, images, and the visualization of results can be turned into a fully collaborative effort. In addition, Jupyter Lab offers a very powerful plugin and extensions system and an application programming interface (API) [81] that allows developers and researchers to add new functionalities to the notebook IDE, making it even more powerful. Particularly relevant to the scope of this paper are extensions meant to make Jupyter Notebooks integrated with classical HPC facilities [82]. At the same time, it is worth highlighting that there are other ways to use notebook in standard HPC settings, like using SLURM [83] interactive sessions and start a Jupyter kernel inside one of them. Thanks to this kind of integrations or solutions, researchers can ensure that resource-intensive calculations can be executed efficiently, expanding the scope of research possibilities while preserving the advantages of using the Jupyter notebook interface.

The final piece of the puzzle is finding a way to share and store Jupyter notebooks within the team and the research community in general. However, simply saving them is not a sufficient target since we also want to preserve the possibility to execute the notebooks. In a nutshell, we want to integrate the Jupyter notebooks with the cloud architecture, while preserving their interactive nature. To this very end, Jupyter Hub was introduced in the Jupyter ecosystem. Jupyter Hub serves as a centralized platform for managing and deploying Jupyter notebooks [84]. It enables multiple users to access shared resources, fostering collaborative research efforts. Jupyter Hub can be particularly advantageous in educational settings, research groups, or institutions where researchers need a centralized hub for their computational chemistry endeavors.

Leveraging all these software products, we can obtain a unified platform for saving and sharing an interactive and multimedia coding environment, which also allows researchers to document and explain their code and research questions. Thanks to the cloud nature of this platform, researchers can save and share their work, and all the editing activity is immediately visible to other researchers. This editing can also be a real-time collaboration between different researchers, further accelerating their activities and the process of getting results. Also, the platform can be developed and deployed following the FAIR principles [85], meaning that all the results and the respective workflows are shared between different teams and are, more generally, freely accessible through the platform. This way, different teams can start from where previous work ended, making it easier to reproduce results but also to re-use previous pieces of research as the starting point of new discoveries. Jupyter has also been used as a tool for sharing computational tasks and workflows [86] to make it easier for researchers to co-operate during the development through a uniform interface [87] and also to build interactive training resources and textbooks [88].

### Workflow management

While Jupyter notebooks are very useful to write and explain the reasoning behind it, they are still far from being a full workflow management solution. Other than being hard to orchestrate and use together in complex pipelines, they still require that researchers write code in order to be built and that they open and read notebooks in order to see if a specific notebook is useful for them. In recent years, low-code approaches are emerging also in the context of research and HPC applications [89]. This approach is particularly appealing as it allows researchers to build even complex workflows and pipelines only using visual tools and connecting functional blocks with logic and temporal order relations.

#### Wireframe sketching

To enhance clarity and structure within computational experiments, the use of wireframe sketches can be invaluable. Wireframes can serve as templates, guiding researchers to structure the workflow of activities systematically. A well-designed wireframe sketch might include sections for input parameters, code execution, visualizations, and textual explanations, promoting consistency and clarity in workflow organization. Wireframes are already a standard tool in software development [90–92], and they are meant to help developers to define the data-flow and execution logic of the software using abstract building blocks and links. Accordingly, wireframes can identify flaws in the general reasoning and improve the logic of the development roadmap. This set of tools can provide computational scientist with systematic ways to better plan the research activities, leaving the implementation work to a later stage. Moreover, this step can benefit from the availability of semantic assets that describe the entities and operations related to research workflows. The actual implementation of a workflow usually follows the complete definition of the generic features in terms of a wireframe sketch. This is when software that is specifically developed in order to give the possibility to implement real-world pipelines with a low-code approach comes to play since it allows to implement a working research flow with a syntax and visual features that are very similar to those of the wireframes.

#### Workflow building tools

Workflow building tools and platforms can assist development and implementation steps starting from wireframe sketches. Workflow builders usually enable the representation of a complex workflow as a sequence of operations connected by sequential and/or logical relationships. The operations are usually represented as blocks or modules, connected to previous blocks via a chain of input/output data structures. The relationships that links these inputs and outputs can be as simple as “after this, do that” or can be more involved and include logical conditions (like: “if this is the output, then do this, or if this is the output, do this instead”). Several general-purpose workflow building platforms have recently gained interest for implementing computational and modelling workflows.

KNIME (Konstanz Information Miner) is an open-source data analytics, reporting, and integration platform [93]. KNIME allows users to visually create data workflows, ranging from simple data preprocessing to complex machine learning and data mining tasks. KNIME provides a graphical interface where users can drag and drop nodes to design and execute data analysis workflows. KNIME employs a node-based workflow design, where each node corresponds to a specific operation or task. Users establish connections between nodes to construct a workflow, allowing data to flow between nodes for diverse operations. The platform boasts an extensive node repository that includes pre-built nodes for tasks like data cleaning, transformation, analysis, and machine learning, giving users the possibility to create custom nodes, thereby expanding the flexibility and the functionality of the platform. Also, KNIME supports the incorporation of data from diverse sources, such as databases, flat files, and web services, providing specific connectors and nodes to ensure smooth data integration and manipulation. Offering high flexibility and extensibility, KNIME allows users to integrate external tools and scripts into workflows, facilitating the inclusion of custom functionalities and algorithms. Moreover, interactive data exploration is facilitated through the provision of interactive views and visualization tools, empowering users to scrutinize and analyze data at various workflow stages. KNIME has also been developed to allow for consistent integration with external tools and languages (with particular focus on popular scientific languages like R and Python), enabling users to harness the capabilities of these tools within the KNIME environment. All these features are further empowered by the community, which developed several extensions and integrations. All these qualities contribute to make KNIME a powerful and user-accessible instrument for the orchestration of workflows and for data analytics in general and to make it widely embraced in both academic and industrial spheres for a diverse spectrum of tasks associated with data manipulation and analysis. KNIME has been used in various nanomaterials research projects for data analysis and workflow automation. For instance, it has been used to develop workflows for the analysis of nanomaterials and nanoparticles toxicity [94] and to aggregate data about biological activities of compounds coming from different sources [95].

The Galaxy Project is an open-source platform designed for accessible and reproducible data-intensive research [96]. While it was conceived for biomedical applications, it is now a more general purpose tool for research workflow automation. Galaxy provides a user-friendly interface facilitating data analysis for scientists, researchers, and analysts. Through a series of integrated tools and workflows, it offers features such as a web-based platform. This web-based interface allows users to access and perform data analysis tasks using a standard web browser, promoting collaboration and ensuring ease of use. Akin to KNIME, Galaxy supports the creation and execution of data analysis workflows. Users can design workflows visually by connecting tools and processes, making it intuitive for researchers with varying levels of expertise. Also, Galaxy incorporates a diverse range of bioinformatics and data analysis tools, consistently integrating them into the platform. Galaxy is designed from the ground up in order to be compatible with various bioinformatics file formats, allowing users to integrate their custom tools, workflows, and results into the platform. Users can then access and execute this plethora of tools within their analysis workflows [97]. By putting strong emphasis on reproducibility in scientific research, Galaxy enables easy sharing of workflows. This feature allows others to reproduce analyses and verify results, fostering transparency and collaboration in scientific endeavors. The Galaxy Project leverages an active community of users and developers and, in general, follows a community-driven approach in order to foster improvement, support, and the development of new features and tools. In addition, Galaxy provides educational resources, tutorials, and training materials to assist users, especially those new to bioinformatics, in getting started with the platform and enhancing their analytical skills. The Galaxy Project is widely utilized in the field of bioinformatics and computational biology, offering a collaborative and user-friendly environment for researchers to conduct data analysis and share their findings with the scientific community.

**Figure 14:**
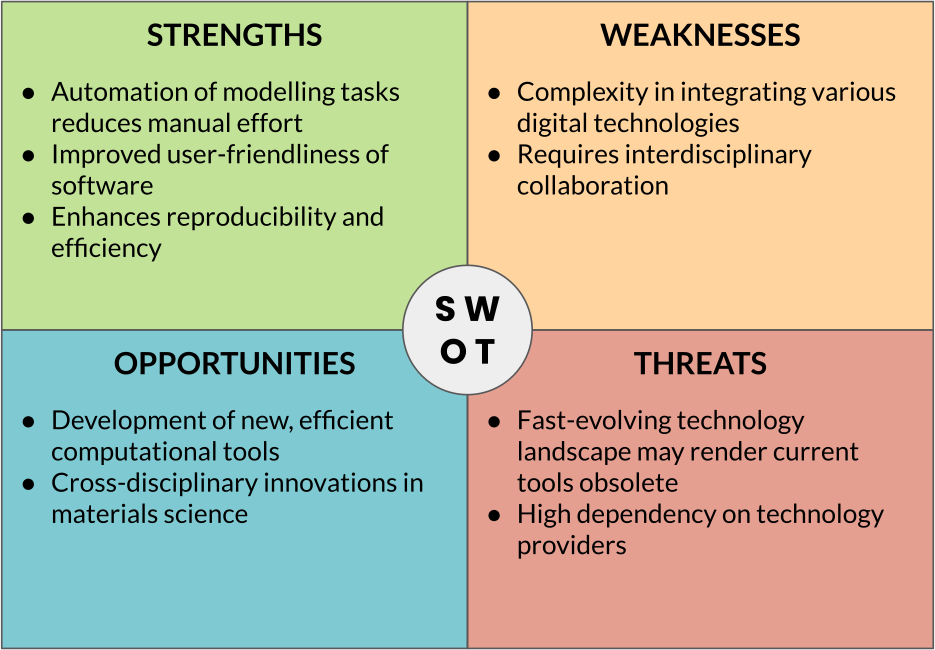
SWOT analysis of the main rising digital technologies and their applications to the materials science domain.

A SWOT analysis related to the technologies discussed in this section is shown in Figure 14.

### Deployment

#### APIs in materials informatics

APIs are standardized sets of protocols and tools that allow different software applications to communicate with each other. They serve as intermediaries, enabling interactions between various systems, applications, and databases. APIs are essential in modern software development, providing the building blocks for creating robust, scalable, and interoperable applications and defining clear methods for requesting and exchanging data, facilitating integration and automation, which are crucial for efficient workflow management. In the context of materials informatics, APIs are gaining increasing importance as they facilitate streamlined data exchange. Thanks to APIs, researchers can automate workflows, access updated datasets, and utilize computational tools without the need for manual data management. This interoperability is crucial for accelerating research by enabling efficient integration of experimental and computational resources. Furthermore, by providing standardized interfaces, APIs ensure that various components of the materials informatics ecosystem can operate together harmoniously, thereby improving the efficiency, reproducibility, and scalability of research processes. In the work of Hu et al. [98], a multialgorithm-based mapping methodology called ChemProps, implemented through RESTful APIs, was proposed to address the inconsistency of polymer indexing due to the lack of uniformity in polymer name expression. Another interesting approach can be found in the work of Hu et al. [99], which proposes the development of MaterialsAtlas.org, a web-based materials informatics toolbox, to address the limited adoption of materials informatics tools due to the lack of user-friendly web servers. This platform includes essential tools for materials discovery, such as composition and structure validity checks, property prediction, hypothetical material searches, and utility tools. MaterialsAtlas.org aims to facilitate exploratory materials discovery by providing accessible and user-friendly tools for materials scientists, thereby accelerating the materials discovery process. The tools are freely available at , and the authors advocate for the widespread development of similar materials informatics applications within the community.

#### Virtualization and containers

Generally, both containerization and virtualization are two of the most widely used techniques when hosting an application on a computer system. Virtualization relies on virtual machines as its essential element, while the fundamental unit of containerization is the container. Clearly, both approaches have advantages and disadvantages. Virtualization involves running an entire guest operating system on a virtual machine, sharing the hardware resources of the physical machine. This introduces a certain overhead, as it is necessary to duplicate the operating system and allocate dedicated resources to each virtual machine. In contrast, containerization can be defined as OS-level virtualization that allows running applications in isolated environments known as containers, sharing the host operating system kernel. Containers are lighter than virtual machines; typically, the startup time of a container is very low, comparable to that of a native application [100–101]. Frequently, containers can run inside virtual machines, and this is one of the most common scenarios encountered when discussing cloud computing. In recent years, multiple containerization technologies have emerged, with Docker [102], Apptainer (formerly called Singularity) [100], and Linux Containers [103] standing out as some of the most utilized and well-known. Docker, in particular, has often become the preferred solution in cloud computing. Singularity was developed with the specific aim of facilitating containerization in the field of HPC. It offers several advantages, notably in terms of use, as it operates without the need for root privileges and lacks daemon processes. Additionally, Singularity provides native support for HPC architectures such as GPUs and Infiniband, enabling simplified communication between different computing nodes. Docker has been already used extensively for making research activities and workflows more easy to reproduce, as shown by recent work [104–106].

#### Orchestration

Container orchestration is the process of automating the majority of operations required to run containerized workloads and services. Specifically, orchestration automates development, management, scaling, and networking of containers. Key orchestration tools, such as Apache Mesos, Docker Swarm, and Kubernetes, provide frameworks for container management. In a typical orchestration tool like Kubernetes, the configuration of an application is described using standard files like YAML or JSON. Once the application specification is planned, the orchestrator assumes various tasks. Primarily, it plans and distributes container resources, makes decisions based on available hardware resources (e.g., CPU, RAM, and storage), and dynamically manages containers in response to workload demands. Network management is crucial, involving the creation of virtual networks for container communication internally and externally through port management. Notably, container orchestration also plays a vital role in data persistence, ensuring storage operations even when a container is recreated. Container orchestration is an essential component for advanced and efficient management of containerized applications in distributed environments. Through orchestration, which coordinates resource distribution, supports horizontal scalability, and manages critical aspects such as network and data persistence, a complex and reliable management system is achieved. Recently, Zhou et al. [107] discussed a novel framework that integrates a resource management layer powered by Kubernetes, demonstrating its application in the field of materials science. This framework leverages Kubernetes for efficient management and orchestration of computational resources. By ensuring dynamic scaling and optimal allocation of both CPU and GPU resources, Kubernetes facilitates job scheduling and execution across heterogeneous computing nodes, significantly enhancing computational efficiency and resource utilization in materials science research.

#### Virtualization and containerization in HPC

Given the significant rise of containers in the development of most common applications, there is a growing consideration for the applicability of containers for HPC. The majority of current containerization implementations rely on Docker and Dockerfile manifests for building container images. However, the direct adoption of container technologies like Docker in an HPC environment proves to be a non-trivial and impractical task, presenting a set of challenges in terms of security and usability that are not easily surmountable. While the use of containers offers an advantage by creating an abstraction layer that simplifies software distribution and management, this abstraction can, in many cases, lead to an increase in required resources and computational effort. A direct consequence of the aforementioned is the emergence of a trade-off within the system software, emphasizing the need for a meticulous and rigorous performance evaluation to identify and quantify the compromises associated with the use of these new container abstractions. HPC clusters are commonly employed for applications demanding low latency and high throughput. However, these clusters are often not inherently equipped to accommodate complex AI workflows along with their specific requirements. Consequently, deploying new packages on such clusters can be challenging for end users. Because of these challenges, containerizing workflows, including intricate simulations integrated with predictive workflows, emerges as an excellent solution. Containerization provides end users with a high degree of customization for their working environment, offering a consistent approach to managing and deploying AI workflows on HPC clusters [108–109].

One of the primary challenges when utilizing conventional HPC infrastructures lies in the fact that jobs are typically managed by a workload manager, which often encompasses diverse responsibilities, including managing the hardware resource limits of the computer cluster, scheduling jobs, ensuring no interference with concurrently running jobs from other users, determining the priority of the different jobs and distributing jobs to available nodes in the most efficient way. As of now, orchestrators such as Kubernetes and others do not possess the capability to fulfill all of these requirements. Consequently, relying solely on containers for cluster utilization proves to be complex. Various works documented in the literature aim to address and overcome these challenges, striving to effectively integrate containers within the HPC environment. Efforts in the literature, such as the study conducted by Keller et al. [110], emphasize specific criteria for HPC container implementations. These criteria include ensuring a secure implementation to safeguard the operating system in multitenant systems, guaranteeing minimal performance overhead, and facilitating optimal system performance through access to vendor-provided libraries and tools tailored for specific HPC hardware. Noteworthy works, including those by Ruiz et al. [111] and Torrez et al. [112], concentrate on the performance analysis within HPC. These studies highlight the gradual improvement in performance over time to cater to the increasing demand for software flexibility in HPC. Through experiments comparing container and bare-metal performance using standard benchmarks, they contribute valuable insights into the evolving landscape of HPC technologies. The extensive efforts documented in the literature to address the challenges of enabling containerized HPC applications, coupled with studies on the integration between orchestrators and workload managers [113–114], underscore the promising trajectory of this technology for HPC configurations. These collective endeavors signify a significant step forward in achieving greater flexibility and efficiency in HPC environments through containerization. A particularly interesting use of containers, especially Docker, can be found in the work of Franco-Ulloa et al. [115], which discusses the development and capabilities of NanoModeler, introducing it as the first webserver designed to automate the construction and parametrization of nanoparticles for molecular dynamics simulations. The NanoModeler Webserver features a frontend built with Angular 6 and Bootstrap for an enhanced, multidevice user experience. The backend utilizes Docker containers, with NodeJS for the orchestrator and data persistence layer.

**Figure 15:**
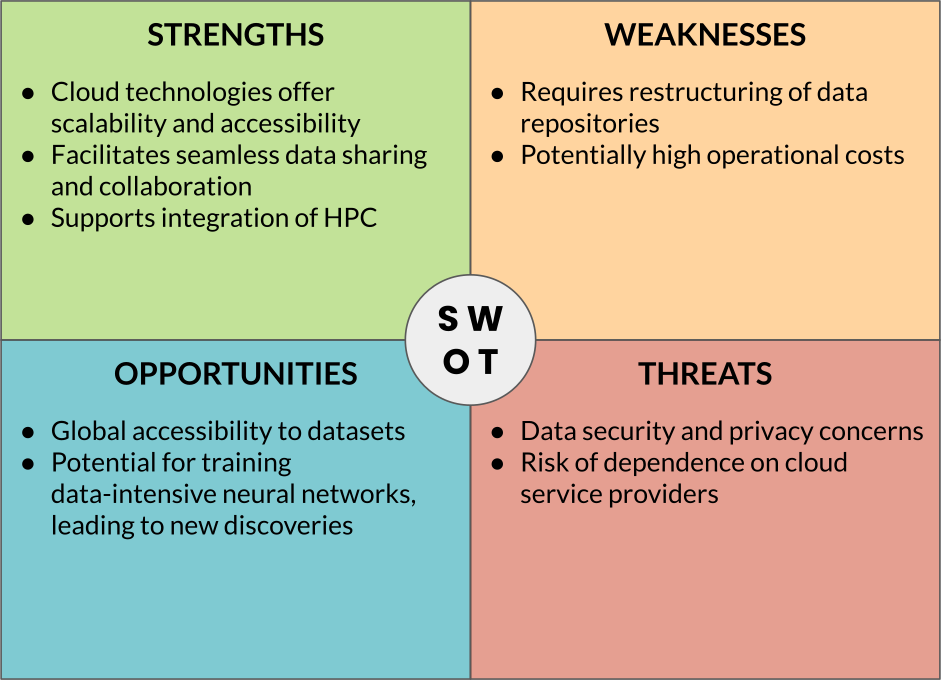
SWOT analysis of infrastructural technologies applied to materials science.

To close this chapter, Figure 15 shows a SWOT analysis applied to the infrastructural technologies.

## Workflows for Property Predictions

If put together, all the techniques and technologies highlighted above can be used to build a general framework that is able to represent and to execute entire research workflows that lead from scientific questions to their answers. Moreover, the workflow and its corresponding results will be semantically linked, improving the reproducibility of the workflow itself and helping in assessing the soundness of the entire pipeline. In addition, the underlying semantics enables us to transform the workflow, the files that we need to perform it, and the final results into actual data that can be stored and retrieved from a database technology and, consequently, used to perform any kind of analysis on them or to train ML models. In the next section, we will analyze a specific case study related to computational workflows in materials and nanomaterials development and illustrate how we envision the future of this approach through the integration of digital technologies.

### Predicting bulk properties of nanomaterials from molecular properties by integrating physical models and ML

In this section, we consider a specific workflow as an example of implementation of the design schemes outlined above. The use case considered consists in the computational modelling of charge transport properties of bulk amorphous molecular materials. Namely, this application represents a typical scenario of multiscale modelling of nanomaterials [116]. This example is partially related to the use case introduced previously when discussing possible applications of the MAMBO ontology. The computational workflow uses the knowledge about the structure of the molecule and a set of procedures to compute the properties of the resulting bulk. The standard workflow considered here is based on the evaluation of the electronic properties of pairs of molecules in aggregates, which are subsequently used in the evaluation of charge transport properties through kinetic Monte Carlo simulations for the whole aggregate. Further details on this approach are given in [117–119].

The whole computational experiment is structured as follows: (i) We start from the information about the structure of a single molecule (for example, a coordinate file in the standard *xyz* format, with Cartesian coordinates and types of atoms). (ii) We perform a set of molecular dynamics simulations on a set of replica of the same molecule within a simulation box. The set of simulations aims at reproducing the amorphous aggregation of molecules within the bulk [2]. At the end of this process, we obtain the morphology of a bulk aggregate. (iii) We extract pairs of molecules from the morphology of the bulk aggregate. To ensure a significant statistical coverage of intermolecular pair configurations, the selection algorithm is biased towards the extraction of pairs with a broad distribution of mutual distance and orientation. (iv) We perform DFT calculations on each molecular pair extracted to compute the electronic coupling. (v) We use the result of the DFT calculations to calculate the charge transfer inside the bulk using kinetic Monte Carlo methods.

As this list clearly shows, this experiment is built using many different computational techniques and requires different information, data structures, and knowledge across different domains and scales. The approach outlined in the previous sections can, therefore, be used to achieve a higher degree of integration across the whole workflow. The resulting integration should lead to significant improvements both in efficiency and in the realization of robust databases and infrastructures. One of the main steps to be undertaken for the implementation of integrated architectures concerns the definition of a shared and unique way to represent all the different tasks of a given workflow in a uniform way. The definition and representation of modular workflow tasks can also support interoperability and the link between different stages of a complex workflow. The development of an ontology, such as MAMBO, can be considered as an ingredient to support the consistent definition of terms and relationships needed to describe a workflow. The example shown in Figure 13 is an example of a possible representation of the content of files containing information on atom positions, encoding the structure of a molecule using different concepts formalized within the reference ontology. Similarly, we can also represent the workflow steps and simulations using the corresponding concepts, thus semantically linking the individual entities and steps to each other. The use of semantic assets to define objects and relationships within the workflow improves efficiency and interoperability and, at the same time, enables modularity. We can then consider to use a workflow building tool to automate the generation of a single executable pipeline. In the example considered, we implemented the workflow within a local instance of the Galaxy platform. Namely, we used both pre-defined blocks made available by the Galaxy community and locally implemented modules. Once the workflow is defined, we can execute resource-intensive tasks on HPC facilities. In the case of Galaxy implementation, we connected the general workflow framework with the underlying HPC infrastructure by using a containerized (Docker) deployment.

In principle, the implementation steps defined above could connect the execution of workflows to centralized databases, enabling the execution of queries. This is where the cloud technologies, if merged with actual database technologies, could give an invaluable contribution to the field. Moreover, these databases can be also realized to enforce the semantic assets defined inside the chosen ontologies to make the queries even more expressive.

The computational workflow defined above, however, exhibits some significant computational bottlenecks. While the generation of the morphology of the bulk molecular aggregate is a relatively quick computation, calculating the electronic coupling for a substantial number of molecular pairs is rather expensive and time-consuming since this computation can require several minutes on a reasonably big HPC infrastructure. Therefore, we also considered the connection of this workflow to ML platforms to increase the overall time-to-solution efficiency. Namely, we computed the electronic coupling only for a limited number of pairs and then used those results to train a ML model for predicting the coupling on the basis of the pair configuration only. Once trained, the ML model is able to predict intermolecular couplings in a few milliseconds on a standard laptop, enabling us to actually compute the electronic coupling for a very large amount of molecules in few minutes. The ML-predicted electronic properties of molecular pairs can then be used to compute the charge transfer in the bulk. We implemented the corresponding tasks within the Galaxy workflow, leading to an efficient and interoperable calculation pipeline. At the end of the entire process, we have a fully automated pipeline, represented as a series of computation blocks and the sequential relations between them, that is able to calculate the charge transfer of a bulk of a molecular materials in a few hours, while having a standardized and logically consistent vocabulary to describe workflow procedures and a unique access point for data.

## Conclusion

In this article, we have explored the profound impact of digital technologies on the realm of materials and nanomaterials, encompassing both experimental and computational research. Specifically, we analyzed the synergies among HPC infrastructures, ML, and data management technologies, elucidating how these interactions empower materials scientists, enhancing the efficiency and reproducibility of their workflows. Additionally, we highlighted the ongoing research into advanced visualization technologies, such as AR and VR, aimed at supporting development in materials science. These technologies offer a promising avenue for designing novel materials and devices by providing intuitive visualizations. The semantic structuring of data emerges as a pivotal capability, facilitating the creation of expansive and comprehensive databases through integrated semantic assets. Leveraging cloud technologies, these datasets become globally accessible, fostering collaboration and facilitating the training of data-intensive neural networks. This, in turn, accelerates investigations into materials properties and expedites the discovery of new materials through enhanced automation. The interconnected nature of these technologies forms a virtuous cycle, each reinforcing and augmenting the capabilities of the others. We showcased our in-house ontology, MAMBO, as an illustrative example of the successful application of such research activities. Notably, software tools such as Jupyter notebooks, KNIME, and the Galaxy Project have significantly eased the interaction with computational infrastructures, lowering entry barriers for researchers and innovators and promoting the reproducibility of research across different areas. Furthermore, the development of tools for building, deploying, and maintaining diverse software components within an HPC facility is crucial. Virtualization and containerization technologies, exemplified by Docker and Apptainer, present promising architectures for managing these intricate systems.

To provide a practical perspective, we introduced a research workflow incorporating various digital technologies, including ML, multiscale simulations, and workflow management. This exemplifies a foundation for the realization of data-driven integration infrastructures, enhancing the efficiency and usability of computational tools. This comprehensive approach has the potential to establish consolidated and shared practices, leading to robust standardization. Ultimately, it enables the implementation of technology transfer pathways for digitalization in nanomaterials development, fostering industrial uptake and paving the way for the future of materials science.

## Data Availability

Data sharing is not applicable as no new data was generated or analyzed in this study.
